# The Inhibition of Folylpolyglutamate Synthetase (folC) in the Prevention of Drug Resistance in *Mycobacterium tuberculosis* by Traditional Chinese Medicine

**DOI:** 10.1155/2014/635152

**Published:** 2014-06-19

**Authors:** Tzu-Chieh Hung, Kuen-Bao Chen, Wen-Yuan Lee, Calvin Yu-Chian Chen

**Affiliations:** ^1^Department of Biomedical Informatics, Asia University, Taichung 41354, Taiwan; ^2^School of Medicine, College of Medicine, China Medical University, Taichung 40402, Taiwan; ^3^Department of Anesthesiology, China Medical University Hospital, Taichung 40447, Taiwan; ^4^Department of Neurosurgery, China Medical University Hospital, No. 2, Yude Road, North District, Taichung 40447, Taiwan; ^5^Research Center for Chinese Medicine & Acupuncture, China Medical University, Taichung 40402, Taiwan; ^6^Human Genetic Center, Department of Medical Research, China Medical University Hospital, Taichung 40447, Taiwan

## Abstract

Tuberculosis (TB) is an infectious disease caused by many strains of mycobacteria, but commonly *Mycobacterium tuberculosis*. As a possible method of reducing the drug resistance of *M. tuberculosis*, this research investigates the inhibition of Folylpolyglutamate synthetase, a protein transcript from the resistance association gene folC. After molecular docking to screen the traditional Chinese medicine (TCM) database, the candidate TCM compounds, with Folylpolyglutamate synthetase, were selected by molecular dynamics. The 10,000 ps simulation in association with RMSD analysis and total energy and structural variation defined the protein-ligand interaction. The selected TCM compounds Saussureamine C, methyl 3-O-feruloylquinate, and Labiatic acid have been found to inhibit the activity of bacteria and viruses and to regulate immunity. We also suggest the possible pathway in protein for each ligand. Compared with the control, similar interactions and structural variations indicate that these compounds might have an effect on Folylpolyglutamate synthetase. Finally, we suggest Saussureamine C is the best candidate compound as the complex has a high score, maintains its structural composition, and has a larger variation value than the control, thus inhibiting the drug resistance ability of *Mycobacterium tuberculosis*.

## 1. Introduction 

*Mycobacterium tuberculosis (M. tuberculosis)* is the principle causative agent in the development of tuberculosis (TB). TB typically attacks the lungs and can be spread from person to person through the air [1] when patients with an active TB infection cough, sneeze, spit, or otherwise transmit respiratory fluids. The mycobacteria may remain latent, causing the host to become weak and maybe develop anorexia. When* M. tuberculosis* becomes active the patients will develop a chronic cough with blood-tinged sputum, fever, night sweats, and weight loss. At this time the disease is infective. In 2012, there were estimated 8.6 million cases of TB worldwide and 1.3 million dead people from the infection (World Health Organization).

Due to the development of drug resistance by* M. tuberculosis*, coupled with the necessity for long-term treatment, it has become difficult to cure this disease by drugs [1, 2]. A recent report in Nature Genetics indicates that the drug resistance genes gyrA, rpoB, rpoC, rpsL, katG, folC, thyA, embB, Rv3806c, and rrs are essential for* M. tuberculosis*. From the above genes, folC and Rv3806c remain uninvestigated [3].

Computer-aided drug design (CADD) is a popular* in silico* simulation technique due to its speed and low cost. The main investigations for CADD are structure-based and ligand-based. In this investigation we use molecular docking and molecular dynamics (MD), two aspects of structure-based drug design, to analyze protein structural variations during the complex interactions [4–9].

Personalized medicine and biomedicine have recently been attracting much attention [10], especially in areas such as the analysis of regional disease [11], clinical diagnoses, disease associated mutations [12]. And, as it is well known throughout the Asian region, traditional Chinese medicine (TCM) is the main personalized medicine resource.

The TCM Database@Taiwan (http://tcm.cmu.edu.tw/) is the world's largest TCM database [13]. In this database the molecular structure and bioactivity of 61,000 TCM compounds are available for screening and many applications of TCM have been identified, such as insomnia treatment [14], pigmentary disorders treatment [15], Parkinson's disease prevention [16], EGFR inhibition [17], inflammation inhibition [18], pain relief [5], and antivirals [19–23]. Today, the screening of TCM compounds from the database is possible by cloud-computing web server [24, 25].

Based on the above research, this study uses the CADD techniques of molecular docking and molecular dynamics to define the protein-ligand interactions and thus reports putative compounds for the inhibition of folC.

## 2. Materials and Methods

### 2.1. Data Collection

The Accelrys Discovery Studio 2.5 (DS 2.5) was used to perform molecular docking. The folC sequence of* M. tuberculosis* was searched on Uniprot (http://www.uniprot.org/, O53174) and the 3D crystal structure (PDB: 2VOS) was download from PDB (http://www.rcsb.org/pdb/home/home.do). The docking site was defined as the dihydropteroate binding site, and thus dihydropteroate was chosen as the control [26].

### 2.2. Disorder Protein Detection

A disordered region of a protein plays an important role in drug design due to the character of the docking site structure affecting the suitability of the complex and the drug efficiency. The floC disorder region could be predicted from the database of protein disorder (DisProt, http://www.disprot.org/) [27], and comparisons between the docking site and the disorder region could help to define the drug effect on the protein [7, 28].

### 2.3. Molecular Docking

Accelrys Discovery Studio 2.5 (DS2.5) software was used to process the molecular docking produced in the CHARMm force field [29] by LigandFit, a receptor-rigid docking algorithm program [30]. The protein transcript from folC has shown that Folylpolyglutamate synthetase, dihydropteroate, and tetrahydrofolate could all dock with the protein. Based on the calculation of Ligplot [31, 32], the complexes formed from the control with the protein product of folC and the top three TCM compounds with the protein product of folC contained hydrophobic interactions.

### 2.4. Molecular Dynamics Simulation

After preparation based on the reference force field [33] of GROMACS 4.5.5 [34] by using SwissParam (http://www.swissparam.ch/) [35], the ligands were subjected to molecular dynamics simulation. The Folylpolyglutamate synthetase with ligands was placed into a simulation box with appropriate buffer, or other solutions, at a minimum distance of 1.2 Å from the complex. The solution for simulation was based on the TIP3P water model in which sodium and chloride ions were added to neutralize complex charges. The MD of GROMACS 4.5.5 had three steps: minimization, equilibration, and production. After minimization with the steepest descent method for 5,000 steps, the structures were transferred for MD simulation. The electrostatic interactions were based on the particle-mesh Ewald (PME) method [36] which calculates each time step at 2 fs and the numbers of steps were repeated 5,000,000 times. Under the 100 ps constant temperature (PER ensemble), the simulation was equilibrated by the Berendsen weak thermal coupling method.

After a MD simulation time of 10,000 ps, the protocols in Gromacs used the MD data to analyze the MD trajectories, RMSD, energy variations, and pathway analysis.

## 3. Results and Discussion

### 3.1. The Detection of Disorder Protein

The disordered protein is intrinsically an unstructured protein, and therefore the docking site will consist of a disordered region that will create challenges for drug docking, and the complex will stabilize only with difficultly. In recent references [7, 28], the disordered protein cannot be established as a common domain; thus a drug docking to a disordered region might have lower side effects. On the other hand, a common domain for a similar structure will allow the drug to dock to the protein easily but may have an effect on other tissues and thus create side effects. This disorder for drug design is not a bad choice and should not be identified as difficult work.

**Figure 1:**
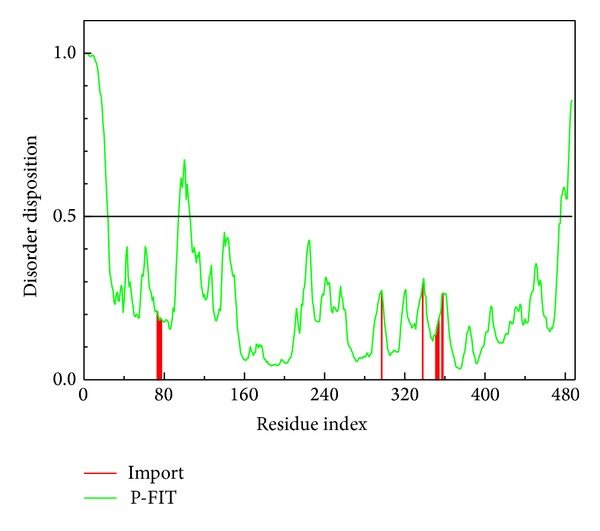
The disorder region prediction and binding site detection. The green curve is the disorder disposition of each amino acid, and the red lines are the residues of the important amino acids.

The important amino acids around the docking site of the synthetase protein are Asn75, Gly76, Lys77, Thr78, Ser79, His299, Asn303, Arg340, Ala354, Ala355, and His356 and are defined as a nondisordered region (Figure 1). From this result, and the understanding of disorder, the compounds and Folylpolyglutamate synthetase could combine as a stable complex.

### 3.2. Molecular Docking

The top three TCM compounds based on the ranking of docking by Discovery Studio 2.5 were selected as candidate compounds for molecular dynamics investigation. These compounds and their botanical sources are listed in Table 1.

**Figure 2:**
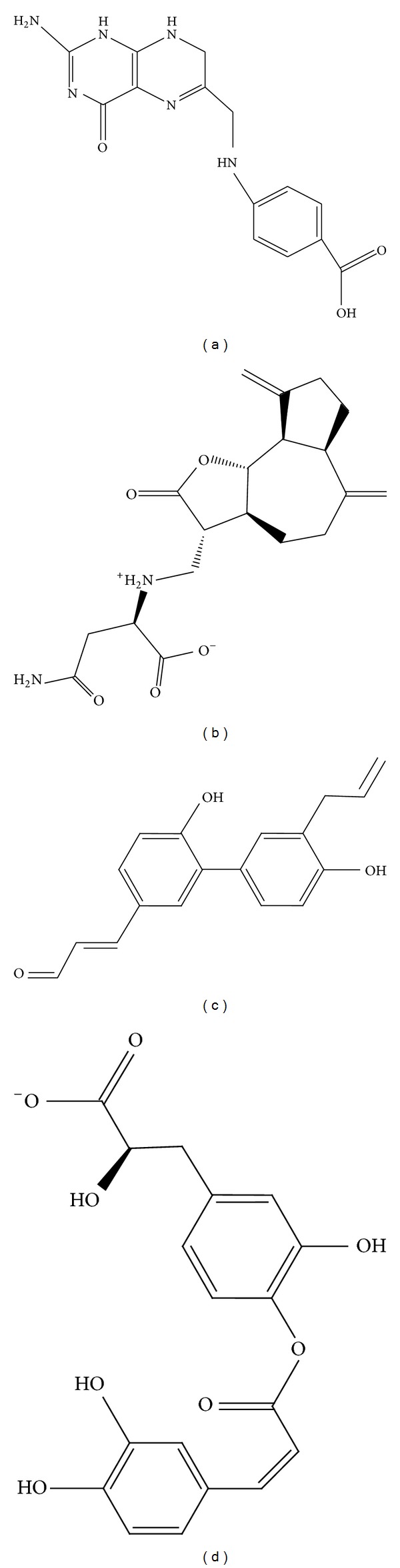
The 2D structure of control and candidate TCM compounds. (a) Dihydropteroate, (b) Saussureamine C, (c) methyl 3-O-feruloylquinate, and (d) Labiatic acid.

The structures of the control drug and the selected compounds Saussureamine C, methyl 3-O-feruloylquinate, and Labiatic acid are presented in Figure 2. The compound with the highest docking score, Saussureamine C, which is extracted from* Saussurea lappa *Clark, is also known as an antiulcer medication [37] and has been used to prevent breast cancer cell migration [38], represses inflammatory responses [39], has antihepatotoxic activity [40], and regulates immunity [41]. The compound ranked second, methyl 3-O-feruloylquinate, derived from* Phellodendron amurense* Rupr., has been assessed for antiviral treatment of H5N1 infections [42], the regulation of fatty acids [43], its role in the protection of human osteoarthritic cartilage [44], the treatment of Alzheimer's disease [45], as an anti-inflammatory [46] and as an antimicrobial, activity against herpes simplex virus type 1 [47], and its effect on the human immune response [48, 49]. The compound ranked third, Labiatic acid, which is derived from* Rosmarinus officinalis* L., has been shown to improve memory impairment [50], as well as having anti-inflammatory activity [51], being able to attenuate oxidative stress and reduce blood cholesterol [52], and having hypoglycemic and hepatoprotective activity [53]. The preceding references indicate these compounds could regulate immunity, be antimicrobial and antiviral and thus may be successful candidate compounds for the inhibition of the activity of bacteria and viruses, and may have the ability to modify drug resistance.

**Figure 3:**
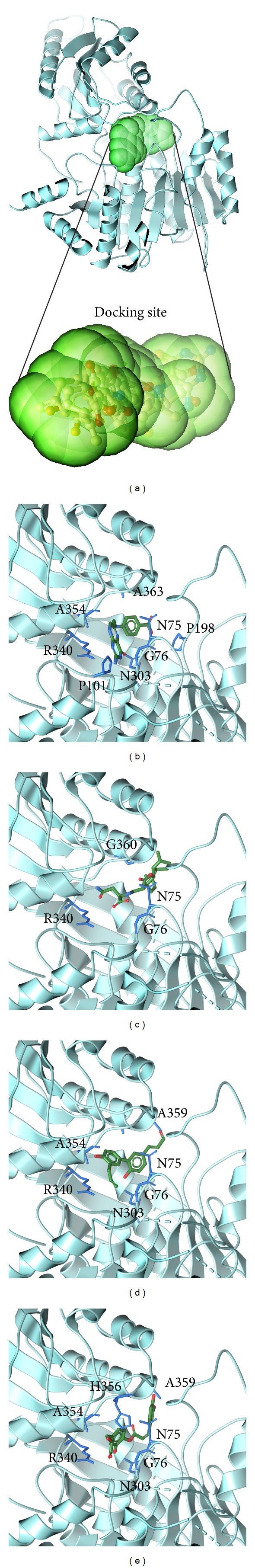
The docking poses of ligands. (a) The crystal structure of Folylpolyglutamate synthetase and the docking site, (b) dihydropteroate, (c) Saussureamine C, (d) methyl 3-O-feruloylquinate, and (e) Labiatic acid.

**Figure 4:**
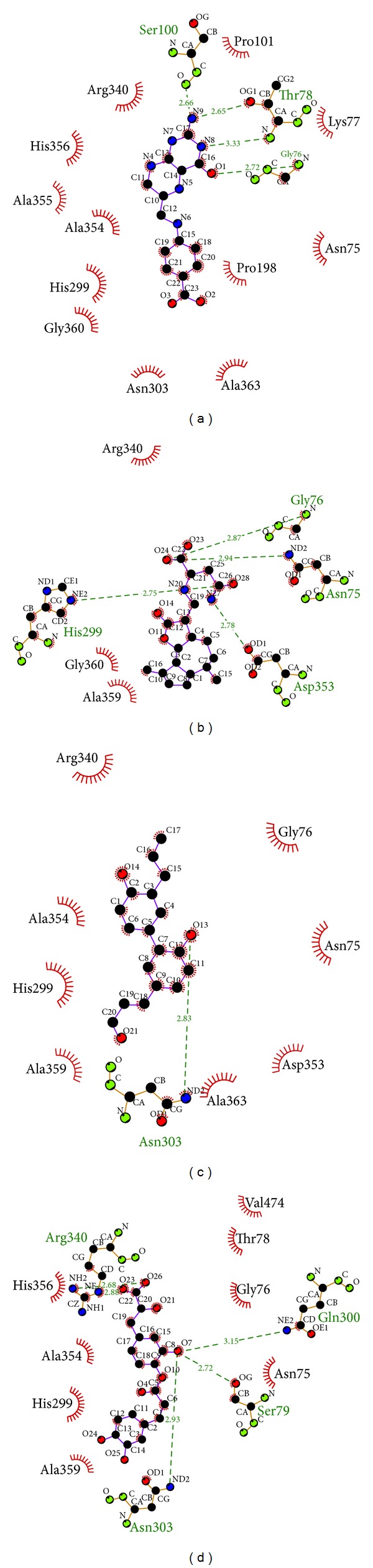
Ligplot illustrations of the hydrophobic interactions. (a) Dihydropteroate, (b) Saussureamine C, (c) methyl 3-O-feruloylquinate, and (d) Labiatic acid. The deep red color of the hydrophobic interactions indicates a high frequency in all ligand interactions.

The docking poses (Figure 3) and hydrophobic interactions (Figure 4) could help with the identification of important amino acids. The results in Figure 3 show the docking poses and the amino acids around docking site that interact with the ligands. The amino acids Asn75, Gly76, Lys 77, Thr78, Ser79, Asn303, Arg340, and Asp353 have been defined in Uniprot as important binding sites. These amino acids are always present during interactions with ligands, not only in docking possess but also in hydrophobic interactions. This result confirms that the docking site is correctly defined as the functional domain of the protein.

### 3.3. Molecular Dynamics Simulation

**Figure 5:**
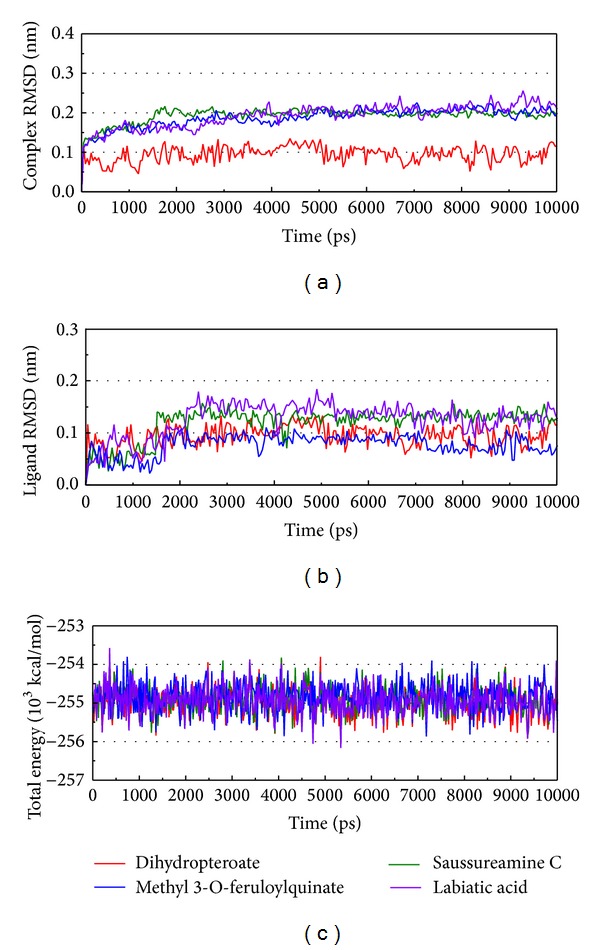
Measures of the MD trajectories. (a) Complex RMSD, (b) ligand RMSD, and (c) total energy.

Variation in the complex RMSD, ligand RMSD, and total energy can help analyze the situation during MD simulation (Figure 5). From Figure 5 it can be seen that the RMSD of the complex and ligand is around, or lower than, 0.2 nm. This result indicates that the protein, ligand, and their complex are stable and that their position and structural variations are not too large. The total energy tends to the range between −254.5 and −255.5 10^3^ kcal/mol. From these results we suggest this simulation will balance quickly according to the stability characteristics of the protein.

**Figure 6:**
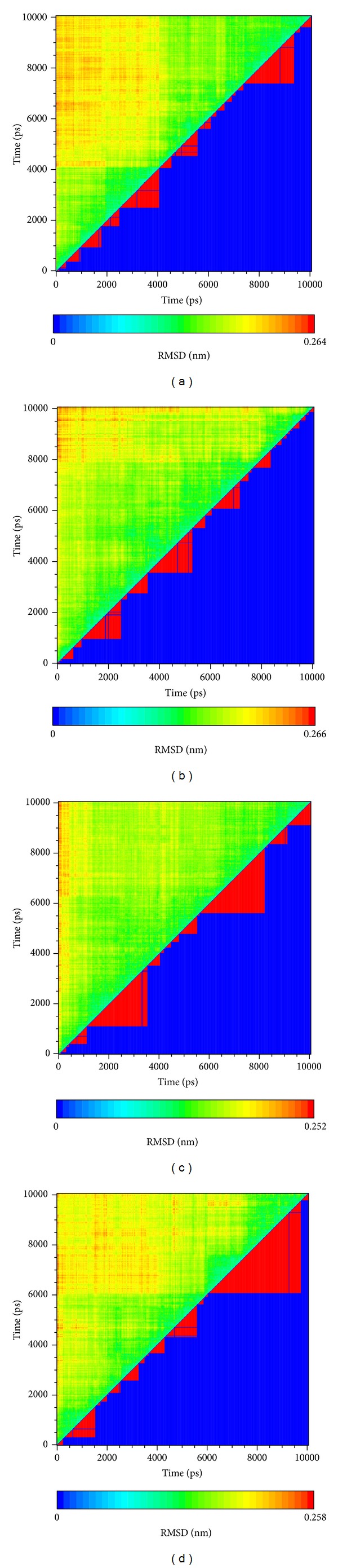
The clustering of RMSD. (a) Dihydropteroate, (b) Saussureamine C, (c) methyl 3-O-feruloylquinate, and (d) Labiatic acid. In the upper triangle the color indicates the RMSD difference between time on the *x*-axis and *y*-axis. Within the lower triangle the red triangles indicate the same group based on similar RMSD.

Clustering assists in grouping the data based on RMSD and thus defines similar structures as belonging to the same group (Figure 6). These results demonstrate that there are some groups which are larger than the primary candidate. This implies that as the simulations tend to balance and thus the complexes have lower variation and similar structure, they become part of the same group. In our previous research we found there were commonly a lot of small groups under 5,000 ps. This interesting situation indicates this protein is stable enough during interactions.

**Figure 7:**
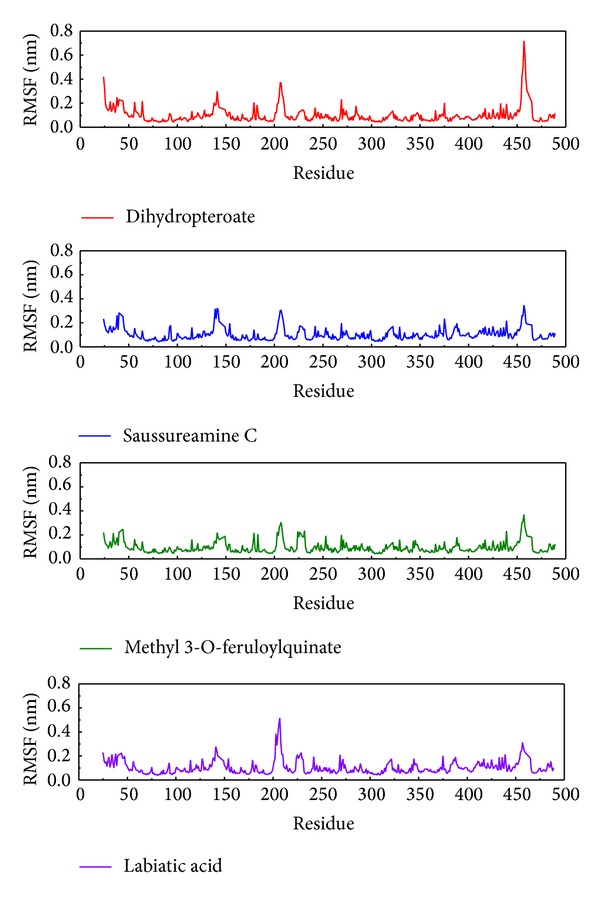
The RMSF detection. For these values of RMSF the curve is the calculation of the RMSD average over the MD simulation, focusing on each residue.

The RMSF calculates the average RMSD focus of each amino acid in the complete MD simulation (Figure 7). In this result, we find the amino acid regions 24, 139–142, 203–208, and 455–460 have large variations. That the defined docking site is not in these regions means that the docking poses will not change significantly while the protein-ligand interactions are mobile. If the RMSF is similar, then the efficacy of compounds may be the same as the control.

**Figure 8:**
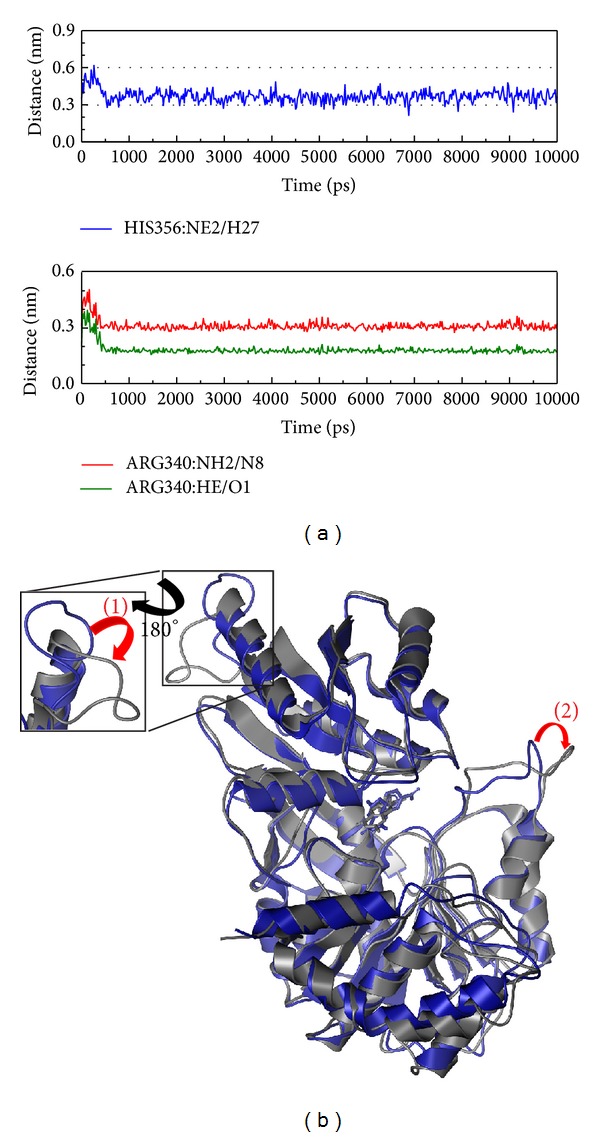
The variation of dihydropteroate and Folylpolyglutamate synthetase complex in MD simulation. (a) H-bond variation and (b) structural variation. The (1)-(2) red color indicates the difference through MD simulation.

**Figure 9:**
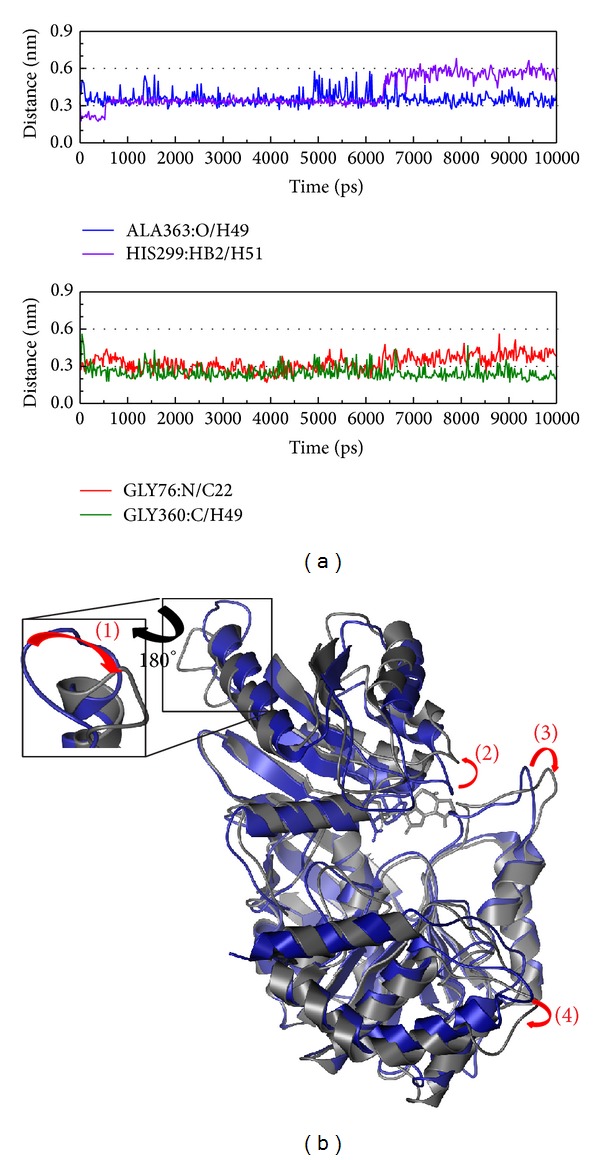
The variation of Saussureamine C and Folylpolyglutamate synthetase complex in MD simulation. (a) H-bond variation and (b) structural variation. The (1)–(4) red color indicates the difference through MD simulation.

**Figure 10:**
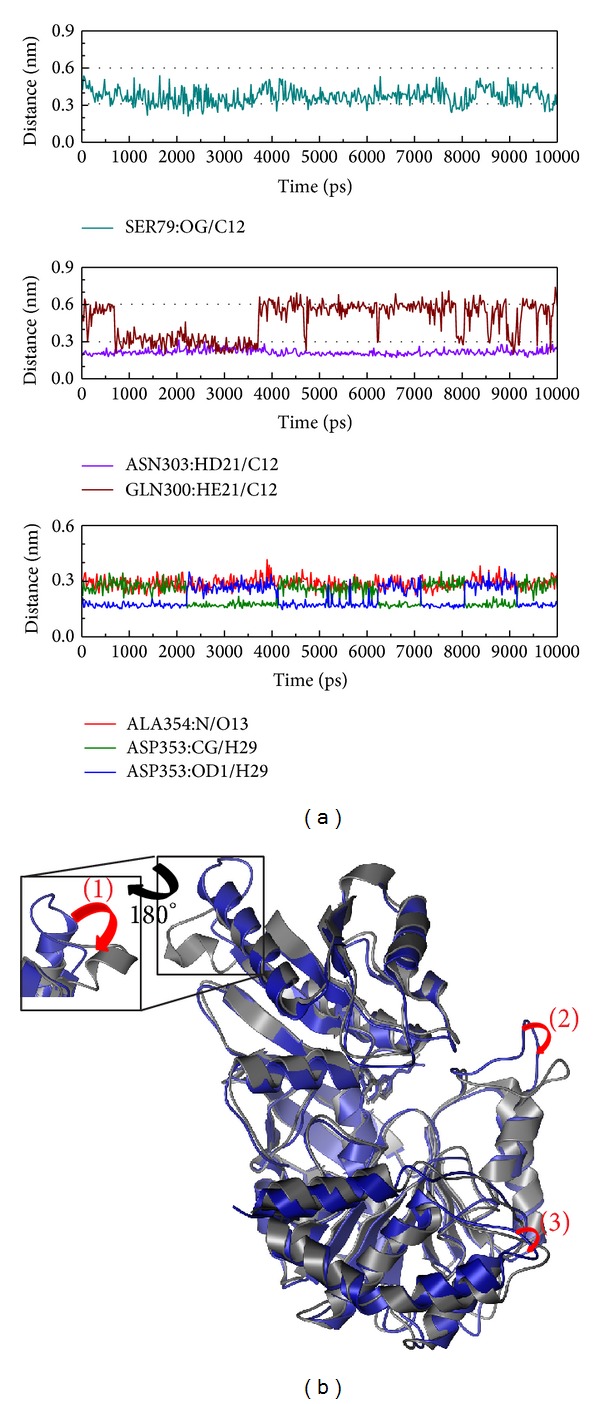
The variation of methyl 3-O-feruloylquinate and Folylpolyglutamate synthetase complex in MD simulation. (a) H-bond variation and (b) structural variation. The (1)–(3) red color indicates the difference through MD simulation.

**Figure 11:**
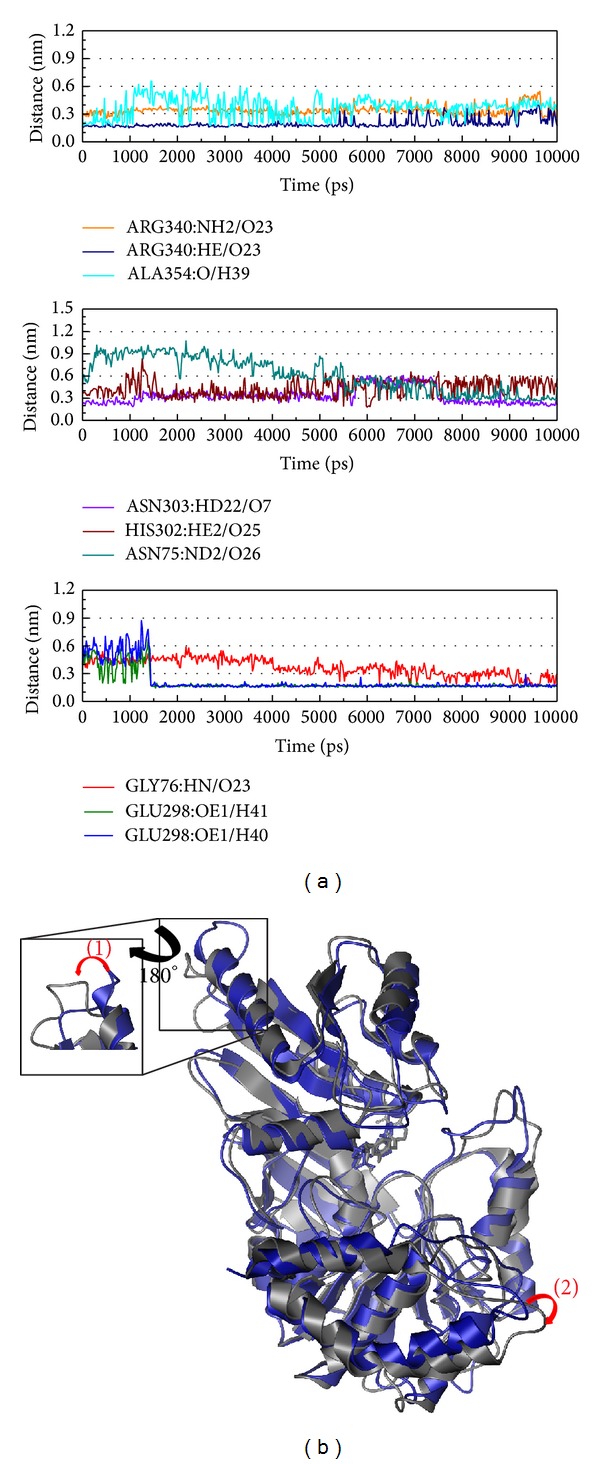
The variation of Labiatic acid and Folylpolyglutamate synthetase complex in MD simulation. (a) H-bond variation and (b) structural variation. The (1)-(2) red color indicates the difference through MD simulation.

Next we discuss the structural variations between protein-control interaction and protein-compound interaction (Figures 8–11).  Figure 8(a) shows that Arg340 formed an H-bond with the ligand (distance <0.3 nm) at 200 ps. This suggests Arg340 may have function in protein-ligand interaction. From Figure 8(b) we can see that the variable region of the protein upper subunit will rotate counterclockwise, while the other subunit rotates clockwise. In the complete protein, there is only positional variation to transform the receptor site for ligand interaction.

In Figure 9(a), His299 produces H-bonds at an early time while Gly360 produces H-bonds at a later time. We suggest His299 might have an effect on the ligand target and Gly360 might have a protein function after the ligand interaction. Similar to the control, the primary candidate compound has the same variation in that the upper subunit rotates counterclockwise and the other subunit rotates clockwise. The variation value in the complex is larger than the control and thus our suggestion is that Saussureamine C might have a stronger effect on the protein.

In Figure 10(a), it is interesting that both Asn303 and Asp353 produce H-bonds during the MD simulation but one of the differences is that the H-bond of Asn303 does not change but the H-bond of Asn303 will exchange two atoms. We think the function of Asn303 may have an effect on the target ligand and that Asp353 may have an effect on the interaction. This complex also has a similar positional variation as the control, but in variation 1 the loop becomes a short helix that might make the protein different.

In Figure 11(a), the H-bond frequency is greater than in other compounds, indicating that Labiatic acid may have a higher activity in this protein. The variation of Arg340 in the MD simulation and Glu298 produce H-bonds from 1,500 ps, indicating that these two amino acids may have a great effect on the protein function. In Figure 10(b), besides the positional variation being similar, variation 1 is present as a short helix loss.

**Figure 12:**
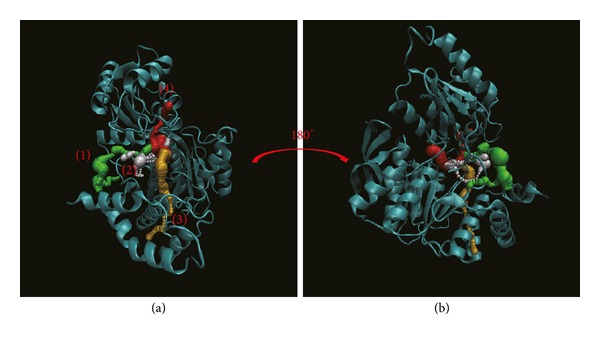
The pathway for dihydropteroate in Folylpolyglutamate synthetase complex. The left picture shows the top 4 pathways for dihydropteroate and then turning 180° from the front to the back is presented in right.

**Figure 13:**
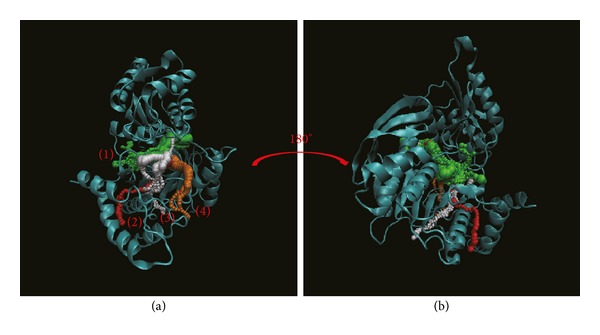
The pathway for Saussureamine C in Folylpolyglutamate synthetase complex. The left picture shows the top 4 pathways for Saussureamine C and then turning 180° from the front to the back is presented in right.

**Figure 14:**
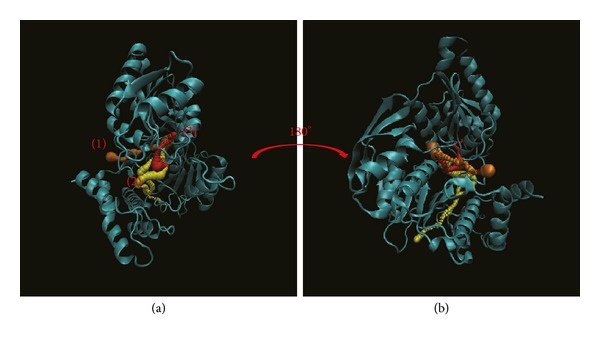
The pathway for methyl 3-O-feruloylquinate in Folylpolyglutamate synthetase complex. The left picture shows the top 3 pathways for methyl 3-O-feruloylquinate and then turning 180° from the front to the back is presented in right.

Pathway definition is based on the calculation of caver 3.0 to find out the path interprotein during MD [54]. These results indicate the different pathways deined form the ligand structure and protein variation caused by interaction (Figures 12 to 14). In Figure 12, this result indicates the top 4 length pathways for dihydropteroate. But in these pathways, the third and fourth are in protein structure not in docking site. Actually, the ligand could not move through protein structure even the range of path could allow ligand pass; thus we suggest pathways 1 and 2 are the true pathways for dihydropteroate. In Figure 13, we also find out the top 4 length pathways in folC for Saussureamine C but we suggest only the first and the fourth are possible pathways. Finally, we can define the first and the third pathways as possible pathways for methyl 3-O-feruloylquinate (Figure 14). In the pathway calculation, there is no pathway for Labiatic acid. We suggest the Labiatic acid makes protein variation; then the path is not larger or longer enough for ligand.

## 4. Conclusion 

In the analysis of docking, this research indicates that the docking site and the ligand dock to protein are correct based on the amino acids interactions. The RMSD, energy, clustering, and RMSF show that Folylpolyglutamate synthetase is a stable protein according to low variation during interaction, with H-bonding providing appropriate assistance. We suggest Glu298, Asn303, Arg340, and Asp353 are important in the interaction based on the high frequency and stability during MD simulation. The structural variation shows that the conformation variation is focused on the protein character rather than the ligand affection. Finally, although the selected compounds are similar to the control in docking, hydrophobic interactions, and structural variations, we suggest that Saussureamine C is the best candidate for the complex as it has a high score, maintains its structural composition, and has a greater variation value than the control.

## Figures and Tables

**Table 1:** Scoring functions of the top three compounds and the inhibitors of folC.

Compounds	Plants	-PLP1	-PLP2	Dock score
Saussureamine C	*Saussurea lappa* Clarke	77.12	69.11	134.058
Methyl-3-O-feruloylquinate	*Phellodendron amurense* Rupr.	94.26	93.3	132.524
Labiatic acid	*Rosmarinus officinalis* L.	89.42	89.08	128.436
Dihydropteroate*		96.11	79.59	29.675

*Control.

## References

[1] Uppuluri R, Shah I (2014). Partial extensively drug-resistant tuberculosis in an HIV-infected child: a case report and review of literature. *Journal of the International Association of Providers of AIDS Care*.

[2] Dos Santos JL, Lima MM, Trindade AB, Carnavalli F, Melchior AC, Chin CM (2013). Tuberculosis: challenges to improve the treatment. *Current Clinical Pharmacology*.

[3] Zhang H, Li D, Zhao L (2013). Genome sequencing of 161 *Mycobacterium tuberculosis* isolates from China identifies genes and intergenic regions associated with drug resistance. *Nature Genetics*.

[4] Huang H-J, Yu HW, Chen C-Y (2010). Current developments of computer-aided drug design. *Journal of the Taiwan Institute of Chemical Engineers*.

[5] Tou WI, Chang S-S, Lee C-C, Chen CY-C (2013). Drug design for neuropathic pain regulation from traditional Chinese medicine. *Scientific Reports*.

[6] Chen CY-C (2013). A novel integrated framework and improved methodology of computer-aided drug design. *Current Topics in Medicinal Chemistry*.

[7] Chen CY-C, Tou WI (2013). How to design a drug for the disordered proteins?. *Drug Discovery Today*.

[8] Basak SC (2013). Recent developments and future directions at current computer aided drug design. *Current Computer-Aided Drug Design*.

[9] Chen CY-C (2009). Weighted equation and rules—a novel concept for evaluating protein-ligand interaction. *Journal of Biomolecular Structure & Dynamics*.

[10] Liao W-L, Tsai F-J (2013). Personalized medicine: a paradigm shift in healthcare. *BioMedicine*.

[11] Lee C-C, Tsai C-H, Wan L (2013). Increased incidence of Parkinsonism among Chinese with *β*-glucosidase mutation in central Taiwan. *BioMedicine*.

[12] Chou I-C, Lin W-D, Wang C-H (2013). Möbius syndrome in a male with XX/XY mosaicism. *BioMedicine*.

[13] Chen CY-C (2011). TCM Database@Taiwan: the world’s largest traditional Chinese medicine database for drug screening In Silico. *PLoS ONE*.

[14] Chen HY, Chang SS, Chan YC, Chen CY (2014). Discovery of novel insomnia leads from screening traditional Chinese medicine database. *Journal of Biomolecular Structure & Dynamics*.

[15] Tang H-C, Chen CY-C (2014). Investigation of the novel lead of melanocortin 1 receptor for pigmentary disorders. *Evidence-Based Complementary and Alternative Medicine*.

[16] Huang H-J, Lee C-C, Chen CY-C (2014). Pharmacological chaperone design for reducing risk factor of Parkinson’s disease from traditional Chinese medicine. *Evidence-Based Complementary and Alternative Medicine*.

[17] Yang S-C, Chang S-S, Chen H-Y, Chen CY-C (2011). Identification of potent EGFR inhibitors from TCM Database@Taiwan. *PLoS Computational Biology*.

[18] Chen K-C, Sun M-F, Yang S-C (2011). Investigation into potent inflammation inhibitors from traditional Chinese medicine. *Chemical Biology and Drug Design*.

[19] Chang S-S, Huang H-J, Chen CY-C (2011). High performance screening, structural and molecular dynamics analysis to identify H1 inhibitors from TCM Database@Taiwan. *Molecular BioSystems*.

[20] Lin C-H, Chang T-T, Sun M-F (2011). Potent inhibitor design against H1N1 swine influenza: structure-based and molecular dynamics analysis for M2 inhibitors from traditional Chinese medicine database. *Journal of Biomolecular Structure & Dynamics*.

[21] Chang S-S, Huang H-J, Chen CY-C (2011). Two birds with one stone? Possible dual-targeting H1N1 inhibitors from traditional Chinese medicine. *PLoS Computational Biology*.

[22] Chen C-Y, Chang Y-H, Bau D-T (2009). Ligand-based dual target drug design for H1N1: swine flu—a preliminary first study. *Journal of Biomolecular Structure & Dynamics*.

[23] Huang HJ, Jian YR, Chen CY (2014). Traditional Chinese medicine application in HIV: an in silico study. *Journal of Biomolecular Structure & Dynamics*.

[24] Tsai T-Y, Chang K-W, Chen CY-C (2011). IScreen: world’s first cloud-computing web server for virtual screening and de novo drug design based on TCM database@Taiwan. *Journal of Computer-Aided Molecular Design*.

[25] Chang K-W, Tsai T-Y, Chen K-C (2011). iSMART: an integrated cloud computing web server for traditional Chinese medicine for online virtual screening, de novo evolution and drug design. *Journal of Biomolecular Structure & Dynamics*.

[26] Sheng Y, Khanam N, Tsaksis Y, Shi X-M, Lu Q-S, Bognar AL (2008). Mutagenesis of folylpolyglutamate synthetase indicates that dihydropteroate and tetrahydrofolate bind to the same site. *Biochemistry*.

[27] Xue B, Dunbrack RL, Williams RW, Dunker AK, Uversky VN (2010). PONDR-FIT: a meta-predictor of intrinsically disordered amino acids. *Biochimica et Biophysica Acta*.

[28] Tou WI, Chen CY (2013). May disordered protein cause serious drug side effect?. *Drug Discovery Today*.

[29] Brooks BR, Brooks CL, Mackerell AD (2009). CHARMM: the biomolecular simulation program. *Journal of Computational Chemistry*.

[30] Venkatachalam CM, Jiang X, Oldfield T, Waldman M (2003). LigandFit: a novel method for the shape-directed rapid docking of ligands to protein active sites. *Journal of Molecular Graphics and Modelling*.

[31] Laskowski RA, Swindells MB (2011). LigPlot+: multiple ligand-protein interaction diagrams for drug discovery. *Journal of Chemical Information and Modeling*.

[32] Wallace AC, Laskowski RA, Thornton JM (1995). LIGPLOT: a program to generate schematic diagrams of protein-ligand interactions. *Protein Engineering*.

[33] Priyakumar UD, MacKerell AD (2005). Comparison of the CHARMM27, AMBER4.1 and BMS nucleic acid force fields via free energy calculations of base flipping. *Abstracts of Papers of the American Chemical Society*.

[34] Hess B, Kutzner C, Van Der Spoel D, Lindahl E (2008). GRGMACS 4: algorithms for highly efficient, load-balanced, and scalable molecular simulation. *Journal of Chemical Theory and Computation*.

[35] Zoete V, Cuendet MA, Grosdidier A, Michielin O (2011). SwissParam: a fast force field generation tool for small organic molecules. *Journal of Computational Chemistry*.

[36] Darden TA, Pedersen LG (1993). Molecular modeling: an experimental tool. *Environmental Health Perspectives*.

[37] Yoshikawa M, Hatakeyama S, Inoue Y, Yamahara J (1993). Saussureamines A, B, C, D, and E, new anti-ulcer principles from Chinese Saussureae radix. *Chemical and Pharmaceutical Bulletin*.

[38] Choi YK, Cho S-G, Woo S-M (2013). *Saussurea lappa* clarke-derived costunolide prevents TNF*α*-induced breast cancer cell migration and invasion by inhibiting NF-*κ*B Activity. *Evidence-Based Complementary and Alternative Medicine*.

[39] Choi H-G, Lee D-S, Li B, Choi YH, Lee S-H, Kim Y-C (2012). Santamarin, a sesquiterpene lactone isolated from *Saussurea lappa*, represses LPS-induced inflammatory responses via expression of heme oxygenase-1 in murine macrophage cells. *International Immunopharmacology*.

[40] Yaeesh S, Jamal Q, Shah AJ, Gilani AH (2010). Antihepatotoxic activity of *Saussurea lappa* extract on D-galactosamine and lipopolysaccharide-induced hepatitis in mice. *Phytotherapy Research*.

[41] Matsuda H, Toguchida I, Ninomiya K, Kageura T, Morikawa T, Yoshikawa M (2003). Effects of sesquiterpenes and amino acid-sesquiterpene conjugates from the roots of *Saussurea lappa* on inducible nitric oxide synthase and heat shock protein in lipopolysaccharide-activated macrophages. *Bioorganic & Medicinal Chemistry*.

[42] Ge F, Ke C, Tang W (2007). Isolation of chlorogenic acids and their derivatives from Stemona japonica by preparative HPLC and evaluation of their anti-AIV (H5N1) activity in vitro. *Phytochemical Analysis*.

[43] Steinmann D, Baumgartner RR, Heiss EH (2012). Bioguided isolation of (9 Z)-Octadec-9-enoic acid from phellodendron amurense ruprand identification of fatty acids as PTP1B inhibitors. *Planta Medica*.

[44] Kim J-H, Huh J-E, Baek Y-H, Lee J-D, Choi D-Y, Park D-S (2011). Effect of Phellodendron amurense in protecting human osteoarthritic cartilage and chondrocytes. *Journal of Ethnopharmacology*.

[45] Xian Y-F, Lin Z-X, Ip S-P, Su Z-R, Chen J-N, Lai X-P (2013). Comparison the neuropreotective effect of Cortex Phellodendri chinensis and Cortex Phellodendri amurensis against beta-amyloid-induced neurotoxicity in PC12 cells. *Phytomedicine*.

[46] Park E-K, Hae IR, Jung H-S (2007). Antiinflammatory effects of a combined herbal preparation (RAH13) of Phellodendron amurense and Coptis chinensis in animal models of inflammation. *Phytotherapy Research*.

[47] Wang W, Zu Y, Fu Y (2009). In vitro antioxidant, antimicrobial and anti-herpes simplex virus type 1 activity of Phellodendron amurense Rupr. from China. *American Journal of Chinese Medicine*.

[48] Mori H, Fuchigami M, Inoue N (1995). Principle of the bark of Phellodendron amurense to suppress the cellular immune response: effect of Phellodendrine on cellular and humoral immune responses. *Planta Medica*.

[49] Mori H, Fuchigami M, Inoue N, Nagai H, Koda A, Nishioka I (1994). Principle of the bark of Phellodendron amurense to suppress the cellular immune response. *Planta Medica*.

[50] Ozarowski M, Mikolajczak PL, Bogacz A (2013). *Rosmarinus officinalis* L. leaf extract improves memory impairment and affects acetylcholinesterase and butyrylcholinesterase activities in rat brain. *Fitoterapia*.

[51] Lucarini R, Bernardes WA, Ferreira DS (2013). *In vivo* analgesic and anti-inflammatory activities of *Rosmarinus officinalis* aqueous extracts, rosmarinic acid and its acetyl ester derivative. *Pharmaceutical Biology*.

[52] Afonso MS, De O Silva AM, Carvalho EB (2013). Phenolic compounds from Rosemary (*Rosmarinus officinalis* L.) attenuate oxidative stress and reduce blood cholesterol concentrations in diet-induced hypercholesterolemic rats. *Nutrition and Metabolism*.

[53] Ramadan KS, Khalil OA, Danial EN, Alnahdi HS, Ayaz NO (2013). Hypoglycemic and hepatoprotective activity of *Rosmarinus officinalis* extract in diabetic rats. *Journal of Physiology and Biochemistry*.

[54] Chovancova E, Pavelka A, Benes P (2012). CAVER 3.0: a tool for the analysis of transport pathways in dynamic protein structures. *PLoS Computational Biology*.

